# Prevalence of nonmedical methamphetamine use in the United States

**DOI:** 10.1186/1747-597X-3-19

**Published:** 2008-07-25

**Authors:** Todd M Durell, Larry A Kroutil, Paul Crits-Christoph, Nina Barchha, David L Van Brunt

**Affiliations:** 1 Eli Lilly and Company, Lilly Research Laboratories, Indianapolis, IN, 46285, USA; 2 RTI International, 3040 Cornwallis Road, Research Triangle Park, NC, 27709, USA; 3 Department of Psychiatry, University of Pennsylvania, 3535 Market Street, Philadelphia, PA, 19104, USA

## Abstract

**Background:**

Illicit methamphetamine use continues to be a public health concern in the United States. The goal of the current study was to use a relatively inexpensive methodology to examine the prevalence and demographic correlates of nonmedical methamphetamine use in the United States.

**Methods:**

The sample was obtained through an internet survey of noninstitutionalized adults (n = 4,297) aged 18 to 49 in the United States in 2005. Propensity weighting methods using information from the U.S. Census and the 2003 National Survey on Drug Use and Health (NSDUH) were used to estimate national-level prevalence rates.

**Results:**

The overall prevalence of current nonmedical methamphetamine use was estimated to be 0.27%. Lifetime use was estimated to be 8.6%. Current use rates for men (0.32%) and women (0.23%) did not differ, although men had a higher 3-year prevalence rate (3.1%) than women (1.1%). Within the age subgroup with the highest overall methamphetamine use (18 to 25 year olds), non-students had substantially higher methamphetamine use (0.85% current; 2.4% past year) than students (0.23% current; 0.79% past year). Methamphetamine use was not constrained to those with publicly funded health care insurance.

**Conclusion:**

Through the use of an internet panel weighted to reflect U.S. population norms, the estimated lifetime prevalence of methamphetamine use among 18 to 49 year olds was 8.6%. These findings give rates of use comparable to those reported in the 2005 NSDUH. Internet surveys are a relatively inexpensive way to provide complimentary data to telephone or in-person interviews.

## Background

Illicit methamphetamine use is a public health concern in the United States with an increase in prevalence in the 1990s. Treatment admission rates for methamphetamine use surged from 10 admissions per 100,000 in 1992 to 52 admissions per 100,000 in 2002 [1]. Prevalence rates have largely stabilized since 2000, although a decrease in new methamphetamine users aged 12 or over occurred between 2004 and 2005 (from 318,000 to 192,000). The 2005 National Survey on Drug Use and Health (NSDUH) found past year and lifetime prevalence rates of 0.5% and 4.3% [2]. About half a million Americans used methamphetamine in the past month [2].

While methamphetamine use in the early 1990s was primarily a problem in the Western United States, the past 15 years have seen it spread across the country. In 1992, 5 states reported high rates of treatment admissions (i.e., >24 per 100,000 population) for primary methamphetamine/amphetamine problems, but by 2002, 21 states reported high rates [1]. Epidemiological data suggest that methamphetamine use in 2005 was highest (0.6% use in past month) among 18 to 25 year olds [2]. From the early to mid 1990s, methamphetamine use doubled among high school students, particularly in Western states [3].

Long-term methamphetamine use is associated with psychiatric symptoms, including drug-induced psychotic symptoms that persist over time [4]. Additionally, in a large sample of treatment-seeking methamphetamine users, 27% had reported a previous suicide attempt, and 43% had reported violent behavior problems [5]. Among methamphetamine-using arrestees in several Western cities, more than a third reported violent behavior as a consequence of their use [6]. Methamphetamine use is also associated with increased risky sexual behaviors [7] and higher prevalence of HIV infection [8,9].

In addition to impacting the individual user, methamphetamine use burdens society with associated criminal justice and health care costs. In 2000, the prevalence of current methamphetamine use among adult male arrestees was approximately 25% in 3 California cities [10]. Methamphetamine use is also highly predictive of self-reported violent criminal behavior and recidivism among parolees, with 82% of methamphetamine users (versus 54% of nonusers) returning to custody within 12 months [11]. Frequent injection of crystal methamphetamine is also associated with high rates of emergency room use [12,13]. The medical cost for treating a patient with severe burns related to methamphetamine production has been estimated to be about $78,000 [14]. Despite little change in prevalence rates since 2000, methamphetamine-related admissions for treatment have increased steadily from 2000 (68,000) to 2004 (129,000). This may be a result of the increased availability (in 2001) of "ice" methamphetamine, a smoked form of highly pure methamphetamine that is thought to be more addictive because smoking gives an intense high [15].

While the NSDUH offers large sample sizes (e.g., 68,305 persons aged 12 or older in 2005 [2]) to provide data on the prevalence of illicit drugs in the United States, other methods may provide comparable data in a more expeditious fashion. Emerging trends can be verified by examination of data from additional studies. If not confirmed, these differences can serve as the basis for further research to explain the different findings.

Telephone surveys cost less than in-person surveys and do not require travel to high-crime areas to interview respondents. However, telephone surveys are increasingly difficult to conduct due to the use of call-screening devices and because these surveys draw their samples from landline telephone numbers. Random-digit dialing (RDD) procedures allow sampling of unlisted telephone numbers and can be designed to increase interviewers' likelihood of reaching eligible numbers. However, about 16% of U.S. households have substituted wireless phone service for landline phones [16].

The use of internet panels is 1 alternative to such time-intensive surveys. In addition to lower cost and increased speed of data collection, internet surveys have the ability to use visual cues to assist in recall [17]. Findings from internet surveys are increasingly being reported in the literature [18].

While internet surveys may yield a sample that is unrepresentative of the target population, propensity methods can address this problem. Weights can be applied by adjusting responses using a propensity scoring method that is tied to the likelihood that an internet survey respondent would have participated in a probability survey. Further statistical adjustments can be made so that the final weights for the internet sample agree with the distribution of the U.S. target population on general demographic characteristics. Nevertheless, because of the concerns about the potential for biases due to respondents self-selecting into an internet survey pool, we stress the importance of having data from a national probability survey as a benchmark against which to compare internet survey findings.

In light of these issues, the current study uses data from a nationwide internet panel to examine the prevalence and correlates of methamphetamine use among adults aged 18 to 49 in the United States.

## Methods

### Study sample and procedures

Subjects were recruited from the Harris Poll Online (HPOL) panel maintained by Harris Interactive. This panel is composed of several million members internationally who have consented to be contacted for public opinion surveys distributed via the internet. Participants were noninstitutionalized civilian adults aged 18 to 49 living in the 50 states and the District of Columbia. Persons younger than 18 were excluded because of the time and costs involved in obtaining parental consent.

The survey was administered and collected in August 2005 through a password-protected, encrypted Web portal. All panel members who initially met eligibility criteria received an e-mail invitation describing the study. These members then received a short screening questionnaire to identify respondents according to specified target group assignments (by age group and substance use history). If a particular target group was not yet filled, eligible members were further directed to a more detailed informed consent form for participation in the full survey. Of the 11,200 HPOL participants who responded to the initial survey invitation, 4,541 were eligible to participate in the study and responded before their respective target groups were filled. This group of eligible respondents included 4,297 (94.6%) members who completed the full survey and 244 (5.4%) members who qualified but refused to participate further. An additional 645 of the 11,200 participants who responded to the initial invitation (5.8%) started but did not finish the full survey.

An Institutional Review Board approved the study procedures prior to data collection. Respondents who completed the survey received a $10 honorarium.

### Measures

Public domain NSDUH questions served as a basis for the substance use and demographic items used in this survey in order to establish comparability between the estimates of the 2 studies. Consistent with NSDUH, respondents were asked about their most recent nonmedical methamphetamine use (defined as drug use without a prescription or to induce a specific feeling or experience), except that the NSDUH category of use "more than 12 months ago" was split into 2 categories: 1) more than 12 months ago but within the past 3 years and 2) more than 3 years ago. From this question, information was obtained about use in the past month, past 12 months, past 3 years, and any time in the respondent's lifetime.

### Statistical analysis

Descriptive statistics (frequency estimates and percentages and related standard errors) were used to estimate population prevalence of methamphetamine use in general and within available demographic subgroups. Weights (see below) were applied to convert raw percentages of respondents to estimates for the civilian, noninstitutionalized population of adults aged 18 to 49 living in the United States. Weighted prevalence estimates and statistical tests were conducted using the SUDAAN Software for Analysis of Correlated Data [19]. The DESCRIPT procedure in SUDAAN was used to conduct *t *tests and generate *p*-values from the tests. Differences between percentages were considered to be statistically significant if the test yielded a *p*-value less than .05. Because analyses were performed on the entire sample, all statistical tests had 4,296 degrees of freedom.

A 2-stage weighting process was used to correct for possible selection bias within the internet panel. First, the data were weighted according to results from a probability-based telephone survey using a propensity scoring approach. The propensity score adjusted for self-selection into the online population and into the panel and for survey nonresponse that may not be explained by demographic differences. The propensity score model was created by Harris using data from parallel telephone and internet surveys that they periodically collect with the telephone survey based on RDD probability sampling. Weights were created so that the weighted distribution of the propensity score for internet respondents was matched to the distribution for the RDD telephone respondents. The second step in the weighting process was to weight the data to match the U.S. target population distribution by general demographic characteristics, as well as distribution of past month cigarette use and past month "binge" alcohol use (i.e., consumption of 5 or more drinks in a single occasion at least once in the past 30 days) estimated from the 2003 NSDUH, the most current publicly available data at the time the study was conducted.

For this study, a model was developed to predict the probability that a response was obtained from a selected individual. For response probability models to be effective, we needed to find covariates that would be related to both (a) the likelihood that a subject was included in the study (i.e., an internet user) and (b) the analysis outcomes being measured in the study. Therefore, the latter 2 substance use measures were chosen because alcohol and cigarette use appear to be related to a respondent's likelihood of being online [20]. Cigarette use and "binge" alcohol use also appear to be related to the likelihood of illicit drug use (2). Further details of the weighting process are given elsewhere [21].

Demographic characteristics of the sample and weighted population percentages also have been described previously [21]. Briefly, the study design for the data set used in this paper favored the inclusion of nonmedical users of prescription drugs used in the treatment of attention-deficit/hyperactivity disorder (ADHD). Consequently, young adults aged 18 to 25 were overrepresented in the sample (77% versus 23% for adults aged 26 to 49). However, the weighting procedures reversed this overrepresentation of young adults to reflect their representation in the population (weighted percentages of 24% for young adults and 76% for adults aged 26 to 49). Persons who were white (and not of Hispanic origin) also were overrepresented in the sample (sample percentage of 77% for whites versus a weighted population percentage of 66%). Males were slightly underrepresented in the sample (43% of the sample versus a weighted percentage of 49%).

## Results

The overall weighted prevalence of lifetime nonmedical methamphetamine use among persons aged 18 to 49 was 8.63% (Table 1). In the past year, the weighted prevalence was 0.71%, and the prevalence of current use (past month) was 0.27%. However, these overall rates masked important differences among subgroups.

**Table 1:** Weighted Prevalence^a ^of Nonmedical Methamphetamine Use by Demographic Characteristics

	**Lifetime**	**Past 3 Years**	**Past Year**	**Past Month**
**Overall**	8.63	2.13	0.71	0.27
	1.50	0.59	0.14	0.09
**Age**				
**18–25**	6.04	3.63	1.79	0.60
	1.04	0.72	0.38	0.22
**26–49**	9.46	1.65	0.36	0.17
	1.94	0.75	0.14	0.09
**Gender**				
**Male**	10.03	3.14	0.86	0.32
	2.04	1.15	0.23	0.14
**Female**	7.27	1.14	0.56	0.23
	2.19	0.29	0.16	0.11
**Education**				
**< High School**	20.64	4.40	0.25	0.02
	7.42	3.25	0.19	0.02
**High School Graduate**	6.12	1.34	0.80	0.21
	1.50	0.36	0.27	0.17
**Some College**	7.31	2.34	0.67	0.31
	1.40	0.64	0.23	0.12
**College Graduate**	5.31	1.41	0.95	0.47
	1.33	0.50	0.33	0.25

^a ^Estimates reflect the percent distributions for each group and their standard errors are displayed below them. Estimates are weighted to the U.S. population of noninstitutionalized civilian adults aged 18 to 49.

Significant differences: Past year use among 18 to 25 years olds > 26 to 49 year olds, *p *= .0004; lifetime use among those without a high school degree > college graduates, *p *= .04; past month use among those without a high school degree < some college, *p *= .01.

Young adults aged 18 to 25 were significantly more likely than older adults aged 26 to 49 to have used methamphetamine in the past year (*t *= 3.53, *p *= .0004). The prevalence of use in the past 3 years did not differ significantly by age group. The lifetime rate was somewhat higher among the 26 to 49 year olds, as might be expected given the greater number of years in which older adults had the opportunity to have used at least once. However, this difference was not statistically significant.

**Figure 1:**
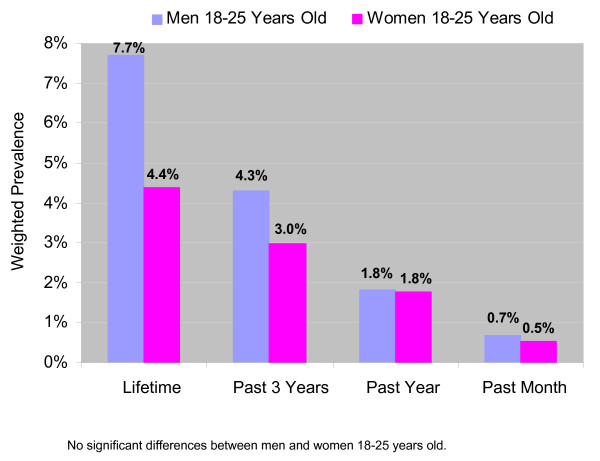
Weighted Prevalence of Nonmedical Methamphetamine Use Among Men and Women Who Were 18 to 25 Years Old.

Prevalence rates did not differ significantly by gender for any period. Within the age group with the highest rates of methamphetamine use (18 to 25 year olds), men and women also had comparable prevalence rates (Figure 1); none of the differences between men and women in this age group were significant.

Those without a high school degree were nearly 4 times more likely to be lifetime methamphetamine users compared with college graduates (*t *= 2.03, *p *= .04). High school graduates and those with some college had similar weighted lifetime prevalence rates, but the rates for both of these groups were not significantly different from the prevalence rate for those without a high school degree. For the past 3 years and past year, the prevalence rates did not vary significantly by education level. In terms of current use, the pattern of use in regard to education was quite different than that seen for lifetime use. There was a trend for increased current use with higher education: 0.21% current use among high school graduates, 0.31% among those with some college, and 0.47% among college graduates. However, these weighted prevalence rates were not significantly different (i.e., *t *= 0.48, *p *= .63 for high school graduates versus adults with some college, *t *= 0.58, *p *= .56 for adults with some college versus college graduates, and *t *= 0.86, *p *= .39 for high school graduates versus college graduates). The weighted prevalence rate for current use among those without a high school degree was very low. Although this estimate also was imprecise (SE: 0.02), it was significantly different from the estimate for those with some college (*t *= 2.44, *p *= .01).

**Figure 2:**
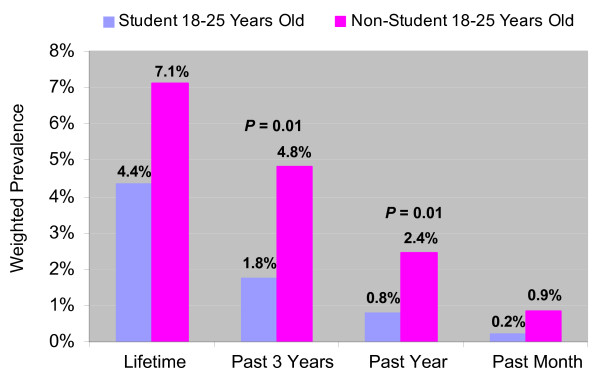
Weighted Prevalence of Nonmedical Methamphetamine Use Among 18 to 25 Years Olds Who Were Students or Non-Students.

As shown in Figure 2, young adults aged 18 to 25 who were not currently enrolled as students were more likely than their counterparts, who were students, to have used methamphetamine in the past year (*t *= 2.51, *p *= .01) and in the past 3 years (*t *= 2.51, *p *= .01). Although non-students in this age group appeared to be about 4 times more likely than students to be current users, this difference was not significant (*t *= 1.65, *p *= .10). Rates of lifetime use also were not significantly different between students and non-students.

**Figure 3:**
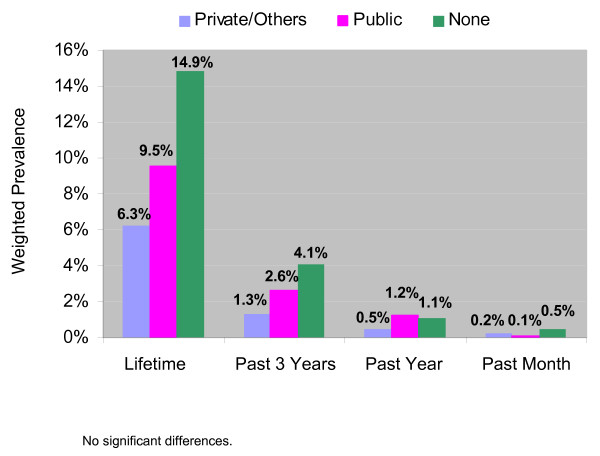
Weighted Prevalence of Nonmedical Methamphetamine Use in Relation to Type of Health Insurance.

Although rates of methamphetamine use in the lifetime and past 3 years appeared to be higher among persons without health insurance compared with persons with private or other insurance (Figure 3), these differences were not significant. There were no significant differences between insurance status groups for methamphetamine use in any period.

## Discussion

Data from this study suggest that at the time of the survey collection (August 2005) 8.6% of the U.S. population aged 18 to 49 had used methamphetamine at least once, and 2.1% had used it in the past 3 years. However, only 0.7% used methamphetamine in the past year, and 0.3% were current users. Other key findings of this survey are 1) the prevalence of past year use was nearly 5 times greater among the 18- to 25-year-old age group than in the 26- to 49-year-old group; 2) methamphetamine use was not exclusively a problem for men, with current use prevalence rates being similar for men and women; 3) non-students within the 18- to 25-year-old age group were more likely than students in this age group to be methamphetamine users in the past year and past 3 years; 4) more than 1 in 5 adults aged 18 to 49 without a high school degree (20.6%) had used methamphetamine at least once in their lifetime; and 5) while uninsured individuals appeared to have elevated lifetime and past 3 year prevalence rates relative to privately insured individuals, there were no significant differences in methamphetamine use, in any timeframe by insurance status.

The 2005 NSDUH, which spanned the timeframe of the current internet survey, reported lifetime rates of methamphetamine use of 4.3% among Americans aged 12 or older and 4.6% among persons aged 18 or older [2,22]. Rates of lifetime methamphetamine use among adult age groups were 5.2% for those 18 to 25, 4.8% for those aged 26 to 34, and 4.5% for those aged 35 or older [22]. Although rates of lifetime methamphetamine use for 18 to 25 year olds, a comparable age group in both studies, were somewhat higher in the present study than in NSDUH, rates in these 2 studies among young adults were similar for the past year and past month.

Demographic correlates of methamphetamine use also were similar in the NSDUH and the present internet survey. Combined data from the 2002 through 2004 NSDUHs [23] and the present study found that past year methamphetamine use was more prevalent in the 18- to 25-year-old age group. In addition, the 2005 NSDUH reported past year methamphetamine use among college students (aged 18 to 22) in 2005 of 0.5% compared to 0.8% for noncollege students in that age range [22]. Although the present internet survey used a slightly different age range (18 to 25), we observed significantly lower rates of past year methamphetamine use among students compared with non-students.

Combined data from the 2002 through 2004 NSDUHs also found a somewhat higher annual average rate of past year methamphetamine use among males aged 18 to 25 (1.8%), compared with 1.4% of females in this age group [23]. The present study also estimated a past year rate of about 1.8% for young adult males but estimated comparable rates among young adults by gender.

In addition, the sample size of the current study (n = 4,297), while still fairly large, may not have had sufficient power to detect statistically significant differences between demographic subgroups for a relatively low-prevalence drug such as methamphetamine. For example, the higher prevalence of current use among 18 to 25 year olds compared with 26 to 49 year olds approached but did not reach statistical significance (*p *= .07). In comparison, there were more than 67,700 completed interviews each year in the combined NSDUH data for 2002 through 2004 [23].

Although data from the 2003 NSDUH were used in the final weight adjustments, methamphetamine use was not 1 of the variables used in the weighting for the present study. Further, the NSDUH results discussed in this article are principally from the 2005 survey. Therefore, the comparability of our findings on methamphetamine use with those from NSDUH bolsters our confidence in our findings based on the use of a large, national, probability survey as a benchmark for calibrating our estimates.

Underreporting of methamphetamine use may be 1 issue for both studies (and for survey measurement of methamphetamine use in general) when questions about use of this substance are asked in conjunction with questions about nonmedical use of prescription drugs. Although methamphetamine can be available in prescription form, most methamphetamine that is now used in the United States is produced illegally in the United States or abroad [23].

In recognition of this issue, the 2005 NSDUH included additional questions to evaluate potential underreporting of methamphetamine use [2]. These new questions were asked in a "noncore" module separate from the "core" prescription stimulant use module; NSDUH includes "core" modules whose content is designed to stay constant across survey years to measure drug use trends and noncore modules that can change from 1 year to the next [2].

When analyses combined positive responses to these noncore questions with data from the core methamphetamine questions, the NSDUH yielded lifetime prevalence rates of 8.4% among 18 to 25 year olds and 6.6% among persons aged 26 or older, compared with rates of 5.2% for 18 to 25 year olds and 5.3% for 26 to 49 year olds based on the core data [2]. In terms of current (past month) use, the 2005 NSDUH estimated a rate of 0.60% among 18 to 25 year olds using their core questions, but this rate increased to 0.83% when the noncore questions were added [2].

This issue also is relevant to the present internet survey because the methamphetamine use questions in this study were taken directly from the core methamphetamine questions in NSDUH and were asked in the context of questions about prescription drugs. Among persons aged 18 to 25, our estimate of lifetime methamphetamine use (6.0% with a standard error of 1.0) fell between the 2005 NSDUH estimate of 5.2% based on the core data and 8.4% based on core and noncore data, though closer to the former. The estimate of current methamphetamine use in this age group (0.6%) was the same as that in the 2005 NSDUH based on the core data. These findings suggest that a similar effect on methamphetamine estimates due to the placement of the methamphetamine questions in the context of questions about prescription drugs may have occurred in the present survey.

We also recognize concerns about biases in the use of a nonprobability sample to make inferences about use of drugs such as methamphetamine in a broader population. In our study, therefore, it was important that we had a nationally representative probability survey such as NSDUH that we could use in weighting our data and as a benchmark against which to compare our results. In particular, comparisons between estimates from the present internet study and the 2005 NSDUH for methamphetamine and other drug use variables that were not used in the weighting procedures suggested that the 2-stage weighting process in the present study yielded estimates that were comparable with those from NSDUH [21]. However, we are not suggesting that internet studies such as this should (or can) replace the need for large, epidemiologic surveys from probability samples. In fact, some surveys, particularly lengthy ones such as NSDUH that cover a wide range of topics and average 1 hour in interview time, would not be acceptable for an internet survey format. Thus, in general, internet surveys should be used in a targeted way to complement rather than replace findings from other data sources.

Keeping in mind the limitations of internet studies, we can suggest that regular internet surveys be considered by states as a methodology for collecting data on methamphetamine or other drug use. At the national level, several ongoing telephone or in-person surveys (e.g., NSDUH, Monitoring the Future) already exist to monitor trends in methamphetamine use. States, however, may find internet surveys to be a promising option for collecting information rapidly and at relatively less cost. The speed and reduced cost of internet surveys may be particularly important when data relevant to a timely policy issue, such as enactment of legislation related to methamphetamine, is required.

The specific cost for internet surveys likely varies with the content and length of the questionnaire, the groups being targeted, the targeted sample size, the length of time required to get the targeted number of interviews, and other factors. The cost advantage of internet surveys lies primarily with the fact that this methodology does not require interviewer labor, which is a key cost component of household surveys that are conducted in-person or via telephone. In these other survey methods, interviewer labor is required for screening sampled units (i.e., households, telephone numbers), recontacting households or individuals in which no one has responded or the sample member is not available, and conducting the interview with sample members who consent to be interviewed. In the current study, the full survey was expected on average to take 20 minutes to complete. Not counting interviewer labor for screening or for multiple recontact attempts, completion of a 20-minute telephone or in-person survey for 4,300 respondents would require more than 1,400 hours of labor just for interviewing.

Another cost advantage for internet surveys is that there are no data entry costs. Other methods of electronic data capture, such as computer-assisted telephone interviewing (CATI) or computer assisted personal interviewing (CAPI), are sometimes used for noninternet surveys. Many studies, however, continue to use paper-and-pencil methods that require considerable time for data entry and checking. In addition, use of CAPI for in-person surveys requires the purchase or availability of a fleet of laptop computers for data collection, and the interviewers need to transmit the data for merging into a data file; in an internet survey, the respondent supplies the necessary hardware and software. Telephone interviews that use CATI would have costs associated with any long-distance calling.

A final cost issue is the ability of an internet survey design to target specific population subgroups to collect information rapidly and efficiently. In the survey that yielded the current data on methamphetamine use, 4,300 responses were obtained in only 3 weeks' time. A telephone survey would likely require months rather than weeks to achieve that number of interviews.

Additional research using national probability samples and focusing on methamphetamine use would be helpful to confirm the current findings. In particular, the current study targeted nonmedical users of prescription medications rather than methamphetamine and used a nonprobability design. Consequently, additional research using probability samples or a future internet survey design targeting methamphetamine users and that is benchmarked to a national probability survey such as NSDUH also could increase the yield of methamphetamine users for further analyses. Given the breadth of topics that surveys such as NSDUH cover, future internet surveys that are calibrated to national probability surveys could be helpful as formative research for generating hypotheses and exploring topics related to methamphetamine use in greater depth.

## Conclusion

This study used an internet panel weighted to reflect U.S. population norms to estimate the prevalence of methamphetamine use among 18 to 49 year olds. The lifetime weighted prevalence was 8.6%, the past year weighted prevalence was 0.71%, and the prevalence of current use (past month) was 0.27%. These findings give rates of use comparable to those reported in the 2005 NSDUH. Internet surveys provide a rapid, relatively inexpensive way of obtaining data that may help inform policy decisions.

## Author Note

Findings that were recently released from the 2006 NSDUH, subsequent to the initial preparation of this manuscript, took into account additional items on methamphetamine that were added to the survey in 2006. These additional items identified respondents who did not report methamphetamine use earlier in the core stimulants module because they did not think of it as a prescription stimulant. Based on these data, estimates of lifetime methamphetamine use in 2006 were 6.42% for young adults aged 18 to 25 and 6.26% for adults aged 26 or older. Respective estimates of past year use for these 2 age groups were 1.69% and 0.61%. The 2006 NSDUH national findings report also presented the results of trend adjustment procedures based on the items from the 2006 survey (see Substance Abuse and Mental Health Services Administration. [2007]. *Results from the 2006 National Survey on Drug Use and Health: National Findings *[Office of Applied Studies, NSDUH Series H-32, DHHS Publication No. SMA 07-4293]. Rockville, MD).

## Competing interests

There are no financial competing interests. The design, methods, and analysis for this study were conducted by RTI International, with funding from Eli Lilly and Company, which is a pharmaceutical manufacturer. RTI received the data collection and analysis contract through a competitive bid. LAK is an employee of RTI International (RTI), an independent nonprofit research firm. Researchers at RTI had full access to all of the data in the study. TD, NB, and DLVB are full-time employees and minor shareholders of Eli Lilly and Company. Paul Crits-Christoph has received consulting income from Eli Lilly, Cephalon, and Alkermes and research grants from the National Institute on Drug Abuse.

## Authors' contributions

TMD, NB, and DLVB assisted with the planning of the study, interpretation of the results, and drafting of the manuscript. LAK participated in the design of the study, collection of data, data analysis, and interpretation of the study and helped draft the manuscript. PC-C assisted with the interpretation of the data and helped draft the manuscript. All authors reviewed and approved the manuscript and provided substantive and methodological input.
